# MMP9/RAGE pathway overactivation mediates redox dysregulation and neuroinflammation, leading to inhibitory/excitatory imbalance: a reverse translation study in schizophrenia patients

**DOI:** 10.1038/s41380-019-0393-5

**Published:** 2019-03-25

**Authors:** Daniella Dwir, Basilio Giangreco, Lijing Xin, Liliane Tenenbaum, Jan-Harry Cabungcal, Pascal Steullet, Audrey Goupil, Martine Cleusix, Raoul Jenni, Abdelwahed Chtarto, Philipp S. Baumann, Paul Klauser, Philippe Conus, Rabindra Tirouvanziam, Michel Cuenod, Kim Q. Do

**Affiliations:** 1 Center for Psychiatric Neuroscience, Department of Psychiatry, Centre Hospitalier Universitaire Vaudois and University of Lausanne (CHUV-UNIL), Prilly-Lausanne, Switzerland; 2 Animal Imaging and Technology Core (AIT), Center for Biomedical Imaging (CIBM), Ecole Polytechnique Fédérale de Lausanne, Lausanne, Switzerland; 3 Laboratory of Cellular and Molecular Neurotherapies, Department of Clinical Neuroscience, CHUV, Lausanne, Switzerland; 4 Service of General Psychiatry, Department of Psychiatry, Lausanne University Hospital (CHUV), Lausanne, Switzerland; 5 Laboratory of Experimental Neurosurgery, Université Libre de Bruxelles, Erasme Hospital, 22, route de Lennik, B-1070, Bruxelles, Belgium; 6 Department of Pediatrics, Emory University School of Medicine, Atlanta, GA USA

## Abstract

Various mechanisms involved in schizophrenia pathophysiology, such as dopamine dysregulation, glutamate/NMDA receptor dysfunction, neuroinflammation or redox imbalance, all appear to converge towards an oxidative stress “hub” affecting parvalbumine interneurones (PVI) and their perineuronal nets (PNN) (Lancet Psychiatry. 2015;2:258–70); (Nat Rev Neurosci. 2016;17:125–34). We aim to investigate underlying mechanisms linking oxidative stress with neuroinflammatory and their long-lasting harmful consequences. In a transgenic mouse of redox dysregulation carrying a permanent deficit of glutathione synthesis (*gclm*^**−/−**^), the anterior cingulate cortex presented early in the development increased oxidative stress which was prevented by the antioxidant N-acetylcysteine (Eur J Neurosci. 2000;12:3721–8). This oxidative stress induced microglia activation and redox-sensitive matrix metalloproteinase 9 (MMP9) stimulation, leading to the receptor for advanced glycation end-products (RAGE) shedding into soluble and nuclear forms, and subsequently to nuclear factor-kB (NF-kB) activation and secretion of various cytokines. Blocking MMP9 activation prevented this sequence of alterations and rescued the normal maturation of PVI/PNN, even if performed after an additional insult that exacerbated the long term PVI/PNN impairments. MMP9 inhibition thus appears to be able to interrupt the vicious circle that maintains the long-lasting deleterious effects of the reciprocal interaction between oxidative stress and neuroinflammation, impacting on PVI/PNN integrity. Translation of these experimental findings to first episode patients revealed an increase in plasma soluble RAGE relative to healthy controls. This increase was associated with low prefrontal GABA levels, potentially predicting a central inhibitory/excitatory imbalance linked to RAGE shedding. This study paves the way for mechanistically related biomarkers needed for early intervention and MMP9/RAGE pathway modulation may lead to promising drug targets.

## Introduction

The pathophysiology of schizophrenia (SZ) was shown to be neurodevelopmental, involving both environmental and genetic factors which converge at redox imbalance and immune dysregulation [1–5]. Although redox balance and regulated immune activation are important for normal brain maturation, uncontrolled oxidative stress (OxS) and neuroinflammation may persistently affect brain development, and more specifically the redox-sensitive Parvalbumin-expressing fast-spiking interneurons (PVI). SZ pathophysiology may involve a “second hit”, i.e., additional environmental insults [6, 7], that leads to increased inflammation and/or OxS, interacting at a critical time during brain development and thereby affecting its normal development in subjects at genetic risk [8–11]. Recent studies suggest that OxS-induced parvalbumin interneurons impairments represents a pathophysiological hub, on which converge various causal genetic and environmental risk factors during neurodevelopment. OxS may result from dysregulation of systems typically affected in SZ, including glutamatergic, dopaminergic, immune, and antioxidant signaling. The genetic vulnerability factors involve either redox regulation genes directly, affecting glutathione (GSH) metabolism and antioxidant defense, or CNVs/genes which indirectly lead to OxS, including 22q11, 15q13, DISC1, PROD, G72, NRG, DTNBP1. Environmental factors known to favor major psychiatric disorders also generate reactive oxygen species (ROS) which, if the redox regulation is impaired, will perturb the developing nervous system [2, 12, 13].

PVI and their enwrapping extracellular matrix, the perineuronal net (PNN), are particularly sensitive to OxS and neuroinflammation [14–16], especially during their maturation period [17–19]. Alterations in PVIs and PNNs, which are both critical for neural synchronization and cognitive functions [20, 21], are a hallmark of SZ [22–24]. However, the mechanisms by which OxS and neuroinflammation affect PVI maturation remain to be elucidated and finding a mechanism that occurs at this vulnerable time-window may pave the way for early-intervention strategies, which are highly relevant for SZ.

Receptor for advanced glycation end-product (RAGE) and matrix metalloproteinase 9 (MMP9) are promising substrates for the interaction between OxS and neuroinflammation, as they are both activated by and mediators of OxS and inflammation [25, 26]. Moreover, alterations in MMP9 [27, 28], in the soluble form of RAGE, and in ligands of RAGE (S100B and AGEs) have been found in the serum of SZ patients [29, 30].

Firstly, we aimed to investigate the molecular mechanism underlying the interaction between OxS and neuroinflammation that leads to PVI/PNN maturation impairments. Then, we explored the long-lasting deleterious consequences of this mechanism on PVI/PNN integrity. Finally, we examined the sensitive period during which it would induce a long-term effect.

In line with the role of OxS as a central hub in SZ pathophysiology, we used a transgenic model of redox dysregulation, the *Gclm* knockout (*Gclm*-KO) mouse, which has a 70% reduction in brain GSH, the major antioxidant in the brain, due to the lack of the glutamate-cysteine ligase (GCL, the key synthesizing enzyme) modulatory subunit (Gclm) [31, 32]. The *Gclm*-KO mice exhibit increased brain OxS as well as morphological and structural anomalies related to SZ phenotypes [31, 33–35]. Specifically, they display spatio-temporal anomalies in PVIs and PNNs in several brain regions including the anterior cingulate cortex (ACC) [36]. In the ACC, maturation of PVIs and PNNs is delayed but is also persistently affected until adulthood when an additional oxidative challenge is applied during the early postnatal period [18], demonstrating a long-lasting deleterious effect of OxS on PVIs.

In a reverse translational train of thought, from model to patients, we explored whether this mechanism can be observed in SZ patients. Moreover, we examined the involvement of this mechanism in the I/E imbalance in early psychosis (EP) patients and more specifically in those with a genetic high-risk genotype for redox dysregulation.

We took advantage of a well-characterized cohort of EP patients that carry GAG trinucleotide-repeat polymorphisms in the GCL gene. In humans, GAG GCL polymorphism alleles containing 8 GAG repetitions (high-risk genotypes) are associated with SZ [9] and lead to a decrease in enzyme expression. This high-risk genotype also predicts low brain GSH levels [37] and increased vulnerability to OxS in fibroblasts [9].

Here, in the *Gclm*-KO SZ model, we highlight the involvement of the MMP9/RAGE pathway in a feedforward loop of OxS and neuroinflammation in early stages of brain development, leading to persistent PVI/PNN damage lasting into adulthood. In EP SZ patients, especially those carrying high-risk GCL GAG genotype, plasma RAGE levels were increased and associated with low prefrontal GABA levels compared to those in healthy controls, pointing to a link between the MMP9/RAGE pathway and I/E imbalance, a hallmark of SZ.

## Materials and methods

### Mice and Treatments

The *Gclm-K*O mice were previously generated by Yang et al. [38] and provided by T. Dalton (University of Cincinnati). *Gclm-*KO and WT mice were subcutaneously injected with GBR-12909 (5 mg/kg, Bio Trend) solubilized in a filtered 0.1 M PBS solution, pH 7.4, for 10 days from postnatal day (PND) 10 to PND 20. The control animals were injected with a filtered 0.1 M PBS solution, pH 7.4, during the same period. To test the hypothesis that MMP9 cleaves Full-RAGE at the membrane, we used a specific MMP2/9 inhibitor named SB-3CT (S1326, Sigma). For the acute treatment, SB-3CT was dissolved in 25% DMSO, 65% PEG-2000 and 10% water at 10 mg/ml, and animals were treated with a dose of 25 mg/kg. *Gclm-K*O and *W*T mice were intraperitoneally (IP) injected at PND 10 or 40 and were sacrificed 2 h or 4 h later by intracardiac perfusion. Control animals were injected with a DMSO/PEG-2000/water as vehicle solution. For the chronic treatment, *Gclm-*KO and WT mice were treated with 25 mg/kg SB-3CT in the same solution as described above from PND18 to PND30 and sacrificed at PND40 or from PND21 to PND33 and sacrificed at PND90. More details on treatments are supplied in Supplementary Information.

### Subject recruitment

Early psychosis patients were recruited from the Treatment and Early Intervention in Psychosis Program (TIPP), a 3-year specialized early psychosis program in the Department of Psychiatry at Lausanne University Hospital, Switzerland [39]. Patients with a duration of illness longer than 5 years were excluded. We considered these patients as “early psychosis” rather than “first episode” as they may have had a previous untreated episode before entering the program.

Healthy controls were matched for sex, age, and smoking status. All assessments (MRS, plasma and clinical) were performed at the same time point (plasma measurement of sRAGE was done on 68 healthy controls and 111 patients, while MRS analysis was conducted on 39 healthy controls and 33 patients). The GAG trinucleotide-repeat polymorphism in *Gclc* gene was genotyped as previously described [9] and assigned into *Gclc* high-risk or *Gclc* low-risk genotype based on the number of GAG repeats as defined in Gysin et al. [9]. More details are provided in Supplementary materials and methods.

### Intracortical injection of siRNA against MMP9

Before surgery, the siRNA solution was prepared using JetSi reagent (Polyplus-transfection, 403-05) and DOPE (L-α-phosphatidylethanolamine dioleoyl, P1223 Sigma) following manufacturer instructions. Then, 1 µl of the siRNA solution was delivered stereotaxically at the following coordinates relative to bregma for injection into the ACC: anterior-posterior (AP) = 0.2, lateral (L) = 0.28, and ventral (V) from the brain surface = 0.2. Animals were sacrificed by intracardiac perfusion 3 days after siRNA injection, as a compromise between the siRNA expression peak and inflammatory recovery from the surgery.

### Intracortical injection of SB-3CT and brain tissue dissection

Since this MMP9 inhibitor has mainly been used in ischemic models when the BBB is known to be leaky, some preliminary experiments were conducted to test the efficacy of SB-3CT and to determine the right dose, by intracortically injecting P40 animals and IP injecting P10 animals. Experimental design and procedure are fully described in Supplementary material and methods.

### Adeno-associated viral vectors

The AAV9-2YF-NRE-eGFP virus was mixed with AAV9-CBA-mCherry at a final titer of 6.5 × 10^12^ and 1.15 × 10^11^ vg/mL, respectively. Surgery was done on PND20 *Gclm*-KO and WT mice with coordinates AP = 0.2, L = 0.8, and V = 2 for 2 ul injection into the right lateral ventricle. Further details are specified in the Supplementary Information.

### Immunohistochemistry

Tissue preparation, immunostaining, and analyses were similar to previous studies but more details are given in the Supplementary Information. For the IH quantification of proteins, the following antibodies were used: anti-RAGE (MAB1179, R&D systems), anti-RAGE (ab3611, Abcam), anti-NeuN (Millipore, MAB377), anti-Iba1 (Abcam, ab5076), anti-CD68 (Abcam, ab53444), anti-CD11b (Bio-Rad, MCA74GA), anti-S100B (Sigma, S2657), anti-MMP9 (Santa Cruz, sc-10737), anti-8-oxoDG (Trevigen, 4354-MC-050), anti-PV (Swant, PV 25), and anti-WFA (PNN) (Sigma, L1516). Images for protein quantification were obtained with a Zeiss LSM 780 Quasar confocal microscope equipped with ×40 and ×20 objectives.

### ELISA for IL-6, IL-1β, and TNFα

IL-6 (eBioscience, 88–7064), IL-1β (eBioscience, 88–7013), and TNFα (eBioscience, 88–7324) ELISA kits were used as specified in the provided instructions. Further details on the procedure are provided in the Supplementary Information.

### ELISA for sRAGE

sRAGE was measured in human plasma samples from patients and controls. In both cases, samples were used undiluted and in duplicate. Human RAGE ELISA (R&D systems, DY1145) kits were used as specified in the manufacturer’s protocol.

### Tissue fractioning and western blot

For tissue fractioning, the specificity of the cytoplasmic and nuclear fractions isolation was tested by blotting anti-alpha-tubuline (TU-02, Santa Cruz) for the detection of cytoplasmic proteins and anti-acetyl-histone H3 (06–599, Millipore) for the detection of nuclear proteins (data not shown). Finally, the two same antibodies used in the IH experiments (anti-RAGE, R&D systems, and antibody anti-RAGE, ab3611, Abcam) were used for detection of Full-RAGE in the cytoplasmic fraction and its intracellular domain in the nuclear fraction. More details on the procedure are provided in the Supplementary Information.

### MMP9 activity

MMP9 activity was measured with the DQ-fluorescein-conjugated gelatin kit (EnzChek® Gelatinase/Collagenase Assay Kit, Life Technology), following the manufacturer’s protocol. More details on the procedure are provided in the Supplementary Information.

### 1H Magnetic resonance spectroscopy (MRS)

All MR measurements were carried out on a 3T MR scanner (Magnetom TimTrio, Siemens Healthcare) with a transverse electromagnetic (TEM 3000) head coil (MR Instruments, Inc). In vivo 1H-MR spectra were acquired from the mPFC [37] using a short-TE spin-echo full-intensity acquired localized single voxel spectroscopy technique (SPECIAL) [40, 41]. More details on the acquisition and analysis are delivered in the Supplementary Information.

### Statistical analysis

For animal data, Student’s *t-*test was used when only two groups were compared, and a two-way ANOVA, when several groups were compared with 2 different factors (e.g., genotype and treatment, or genotype and age), followed by a Tukey post hoc test for multiple comparison tests. For human analysis, the influence of different factors on the sRAGE levels was tested using a linear regression model. The correlation between 2 different parameters was tested with a correlation test. All statistical analyses were conducted on R studio software. More details are supplied in the Supplementary Information.

## Results

### Oxidative stress induces microglia activation during early development

**Fig. 1:**
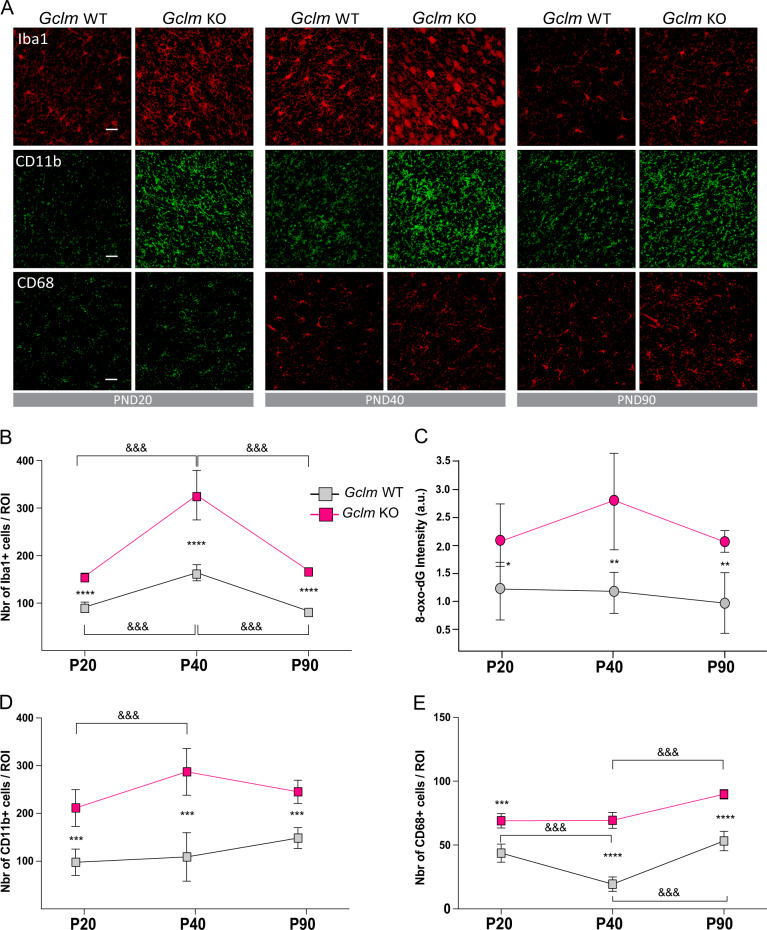
Microglia activation is higher in the ACC of Gclm-KO mice than in the ACC of WT mice throughout development. **a** Confocal images (Scale bar: 30 μm) showing Iba1, CD11b, and CD68 staining in the ACC of Gclm-KO and WT mice at PND20, PND40, and PND90. Graphs represent the quantification of Iba1+ (**b**), 8-oxoDG intensity (**c**), CD11b+ (**d**) and CD68+ (**e**) cells at PND20, PND40, and PND90. **b**–**e** Data are expressed as the mean ± s.e.d. (*n* = 5–6). Analyzed by 2-way ANOVA with 2 factors (genotype and age) followed by Tukey post hoc test. For genotype effect: ****P < 0.0001; ***P < 0.001; **P < 0.01; *P < 0.05; For age effect: &&& *P* < 0.001

**Fig. 2:**
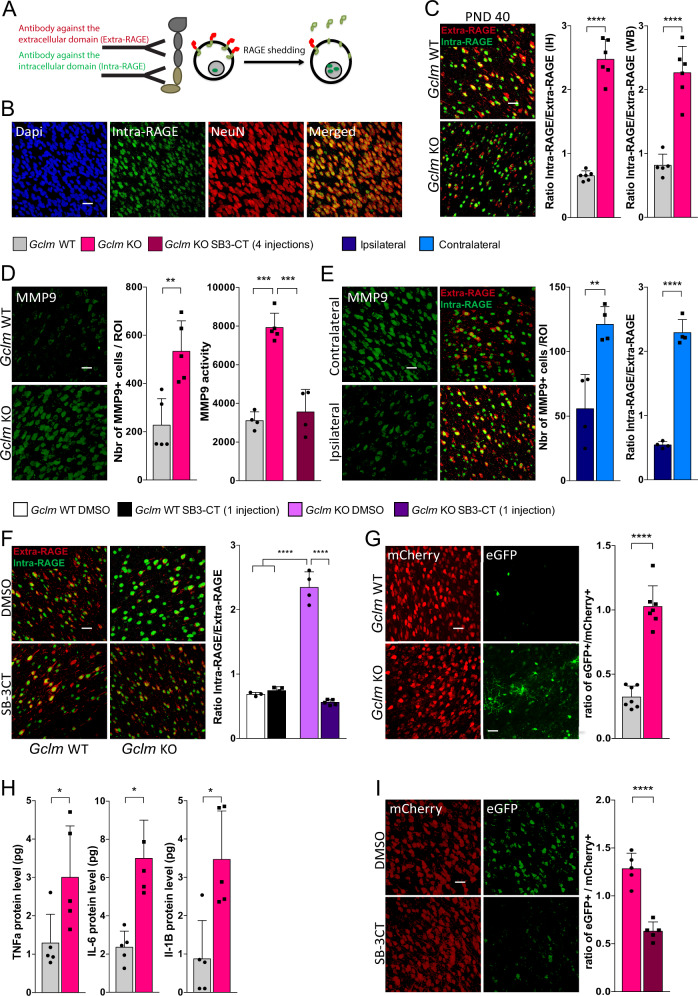
RAGE shedding, induced by MMP9, NFkB activation and pro-inflammatory cytokines are increased in the ACC of Gclm-KO mice as compared to WT mice at an early stage of development. **a** Schematic representation of the IH strategy for RAGE shedding visualization using two different antibodies. **b** Confocal images (Scale bar: 30 μm) showing RAGE intranuclear domain co-localization with NeuN, a neuronal marker. **c** RAGE shedding is higher in the ACC of Gclm-KO mice than in the ACC of WT mice at PND40. Confocal images (Scale bar: 30 μm) of RAGE shedding (expressed as a ratio of Intra-RAGE over Extra-RAGE) with the corresponding quantification graph for PND40. WB quantification of Intra-RAGE in the nucleus over Extra-RAGE at the membrane, using the fractioning method. **d** MMP9 protein/activity are higher in the ACC of Gclm-KO mice than in the ACC of WT mice at PND40. Confocal images (Scale bar: 30 μm) of the MMP9 protein level with corresponding quantification graph and a graph showing the MMP9 activity measured in the ACC by gelatin zymography in Gclm-WT and KO mice. SB-3CT (25 mg/kg) was injected 4 times, each injection separated by 4 days, from PND18 until PND30. **e** Inhibition of MMP9 by intracortical siRNA injection prevents RAGE shedding in the ACC of Gclm-KO mice. Gclm-KO mice were sacrificed 3 days after siRNA injection. Confocal images (Scale bar: 30 μm) showing MMP9 and RAGE shedding and the corresponding graphs showing the quantification at the site of injection (ipsilateral) and the contralateral, uninjected site. **f** Acute inhibition of MMP9 by peripheral injection of SB-3CT prevents RAGE shedding in the ACC of Gclm-KO. Confocal images (Scale bar: 30 μm) showing RAGE shedding in Gclm-KO and WT mice injected IP with SB-3CT (25 mg/kg) and sacrificed 2 h later. **g** NFkB activation is increased in the ACC of Gclm-KO compared to the levels in the ACC of WT mice at PND40. Gclm-KO and WT mice received intraventricular injection of AAV9-2YF-NRE-eGFP and AAV9-CBA-mCherry viral vectors at PND20 and were sacrificed at PND40. Confocal images (Scale bar: 30 μm) of the eGFP and mCherry signal and corresponding quantification of the eGFP+/mCherry+ cell ratio. (**h**) Pro-inflammatory cytokines are increased in the ACC of Gclm-KO compared to the levels in the ACC of WT mice at PND40. Quantification of the TNFα, IL-6, and IL-1β protein level by ELISA in the PFCx of Gclm-KO and WT mice. **i** NFkB activation is prevented by MMP9 inhibition with SB-3CT in the ACC of Gclm-KO mice at PND40. Confocal images (Scale bar: 30 μm) of the eGFP and mCherry signal and corresponding quantification of the eGFP+/mCherry+ cell ratio at PND40 after 4 injections of SB-3CT (25 mg/kg), each injection separated by 4 days, from PND18 until PND30. **a**–**i** Data are expressed as the mean ± s.e.d. (*n* = 4–8). ****P < 0.0001; ***P < 0.001, **P < 0.01; *P < 0.05; analyzed by Student’s *t*-test (**a**, **d**, **e**, **g**, **h**, **i**) or 2-way ANOVA followed by Tukey post hoc test (**d**, **f**)

*Gclm*-KO mice have previously been shown to express higher levels of OxS markers than wild-type (WT) mice in the ACC from an early postnatal age (postnatal day (PND)20) until adulthood (PND90) [18]. Therefore, we investigated microglia activation in the ACC during the same period (PND20-90). Microglia activation was assessed using three different markers: (1) Iba1 to label all microglia, (2) CD11b to label phagocytic microglia, and (3) CD68 to label non-phagocytic amoeboid microglia. There were significantly more Iba1-immunoreactive (+), CD11b+ and CD68+ cells in the ACC of *Gclm*-KO mice at PND20, 40 and 90 than in that of the WT (Figs 1, 2-way ANOVA with multiple comparison tests). In contrast, neither microglia activation nor OxS was significantly increased in the somatosensory cortex of *Gclm*-KO mice [18] (Suppl. Figure 1B), suggesting a causative effect of OxS on microglia activation. Since astrocytes, together with microglia, are involved in the neuroinflammatory response, we quantified the number of cells immunoreactive for S100B, a specific astrocytic pro-inflammatory marker. In line with the microglia activation results, there were also more S100B+ cells in *Gclm*-KO mice than WT mice at PND40 (Suppl. Figure 1A).

Since microglia are known to play an important role during early maturation, microglia activation was studied at different postnatal developmental stages. Interestingly, the Iba1+ cell number was high in early postnatal days, peaked in the peripubertal stage (PND40) and then decreased in adulthood (PND90) in both genotypes, in parallel to the OxS dynamics measured by a marker of mitochondrial DNA oxidation (8-oxoDG) (Fig. 1b, c). Moreover, morphological observations of Iba1+ cells in *Gclm*-KO mice at PND40 revealed the presence of amoeboid microglia, which were not detected in the WT (Fig. 1a). The amoeboid morphology is indicative of phagocytic microglia, which, at this peripubertal age, are associated with synaptic pruning [42]. At PND90, some residual amoeboid microglia were found in *Gclm*-KO but not WT mice (Fig. 1a).

Thus, OxS-induced by redox dysregulation led to neuroinflammation, which was particularly prominent in early development (PND40).

### MMP9 activation by oxidative stress leads to increased RAGE shedding

RAGE has been associated with pro-oxidant and inflammatory conditions, and its shedding, cleavage of RAGE at the membrane (Full-RAGE) releasing its extracellular domain (Extra-RAGE) that is secreted (soluble RAGE) and its intracellular domain (Intra-RAGE), has been demonstrated in several in vitro studies [43]. However, RAGE shedding is poorly characterized in tissue in vivo as its soluble form (sRAGE) is only measurable in biological fluids [44]. To investigate its potential role in OxS-susceptible *Gclm*-KO mice, we used two antibodies simultaneously, one targeting the Extra-RAGE and the other recognizing the Intra-RAGE (Fig. 2a), thus allowing for visualization and quantification of the shedding process. By immunohistochemistry (IH), we found Extra-RAGE on the membrane and Intra-RAGE in the nucleus, specifically in neurons (Fig. 2b).

RAGE shedding (the ratio of the number of cells expressing Intra-RAGE/ the number expressing Extra-RAGE) was significantly higher in neurons of *Gclm*-KO mice than in those of WT mice at PND20 and at PND40 but not at PND90 (Fig. 2c, Suppl. Figure 2B). At PND40, Western blot (WB) quantification of Intra-RAGE in the nuclear fraction and Extra-RAGE in the cytoplasmic fraction further confirmed the difference in RAGE shedding on brain tissue with the fractioning method (Fig. 2c).

The shedding process has been described as involving a disintegrin and metalloproteinase domain-containing protein 10 (ADAM10) and/or MMP9 [43, 45] in vitro. As MMP9 contains a redox-sensitive SH-SH switch at its active site [46] (Suppl. Figure 2A), we explored whether the MMP9 protein level and activity was higher in *Gclm*-KO mice than in WT. Neurons in *Gclm*-KO mice expressed higher levels of MMP9 than those in WT mice at PND20, 40 and 90 (Fig. 2d, Suppl. Figure 2C). Furthermore, MMP9 activity in the ACC of *Gclm*-KO mice was also increased at PND40 compared to that in WT mice, measured by gelatin zymography (Fig. 2d). Interestingly, the MMP9 level peaked at the peripubertal stage (PND40) and then decreased markedly in adulthood (PND90) (Suppl. Figure 2C).

The potential involvement of MMP9 in RAGE shedding was assessed by evaluating RAGE shedding 3 days after (PND40) injection of an siRNA targeting MMP9 into the ACC of *Gclm*-KO mice at PND37, allowing time for siRNA expression. Indeed, the MMP9 protein level in the injected side was decreased by the specific siRNA (54 ± 21%) compared to that in the uninjected side (Fig. 2e). Subsequently, RAGE shedding was decreased following MMP9 downregulation (Fig. 2e), demonstrating the critical role of MMP9 in RAGE shedding.

To exclude the potential pro-inflammatory effect of the surgery, a specific inhibitor of MMP2/9, SB-3CT, was systemically injected. This molecule has been shown to bind directly to the zinc atom in the catalytic site of MMP9, interfering with its activation [47]. It can cross the blood-brain-barrier (BBB) [48] and can prevent the tissue damage and remodeling induced by MMP9 [49]. After validation of this inhibitor for peripheral use (Suppl. Figure 3), either the SB-3CT solution (25 mg/kg) or a control DMSO/PEG-2000 solution was injected intraperitoneally (IP) into *Gclm*-KO and WT mice at PND40, and RAGE shedding was quantified 2 h later. While the inhibitor had no effect on the WT mice, RAGE shedding was prevented by systemic injection of the MMP9 inhibitor in the *Gclm*-KO mice compared to the shedding measured in the *Gclm*-KO mice treated with DMSO/PEG-2000 (Fig. 2f), consistent with the siRNA results.

Thus, these findings confirm the key involvement of MMP9 in RAGE shedding in the ACC of *Gclm*-KO mice at PND40.

### Increased NF-kB activation and pro-inflammatory cytokines in the ACC of *Gclm*-KO mice at PND40

A major component of neuroinflammation is the activation of nuclear factor-kB (NF-kB), which induces pro-inflammatory factors. Full-RAGE, located at the membrane, has been shown to activate NF-kB [50], and MMP9 is known to induce neuroinflammation through different pathways [51]. As the effect of RAGE shedding on NFkB activation remains unknown, we investigated this effect in the *Gclm*-KO at PND40. Instead of using antibodies targeting activated subunits of NFkB, which lack specificity and sensitivity, we took advantage of the adeno-associated virus (AAV) technologies to measure NFkB activation. An AAV containing a plasmid expressing eGFP under the control of repeated NFkB responsive elements (NREs) was developed to specifically reveal NFkB activation in vivo [52]. This vector was encapsidated into a variant serotype 9 capsid (AAV9-2YF) previously shown to enhance transduction efficiency in the brain [53]. The AAV9-2YF-NRE-eGFP was injected into the ventricle of *Gclm*-KO and WT mice to allow a widespread diffusion of the virus. As a reference for the virus diffusion, an AAV9 containing a plasmid expressing mCherry under a strong and constitutive promoter (the hybrid chicken-beta-actin/CMV or “CBA” promoter) was co-injected with the AAV9-2YF-NRE-eGFP virus. The eGFP+ cells were quantified in the ACC of PND40 *Gclm*-KO and WT mice and normalized to the number of mCherry+  cells (ratio eGFP/mCherry). Importantly, the serotype of the virus ensured that the eGFP+ expressing cells were neurons [54]. The eGFP+ cell number was markedly higher in the *Gclm*-KO mice than in the WT, reflecting increased NF-kB activation (Fig. 2g). Consistent with NF-kB activation, the levels of interleukin (IL)-1β, tumor necrosis factor (TNF)α, and IL-6 were all higher in *Gclm*-KO than in WT mice (Fig. 2h), reinforcing the increased neuroinflammatory state in the *Gclm*-KO mice at an early developmental stage (PND40).

### Inhibition of MMP9 early in development prevents the oxidative stress/neuroinflammation-induced decrease in PNNs

**Fig. 3:**
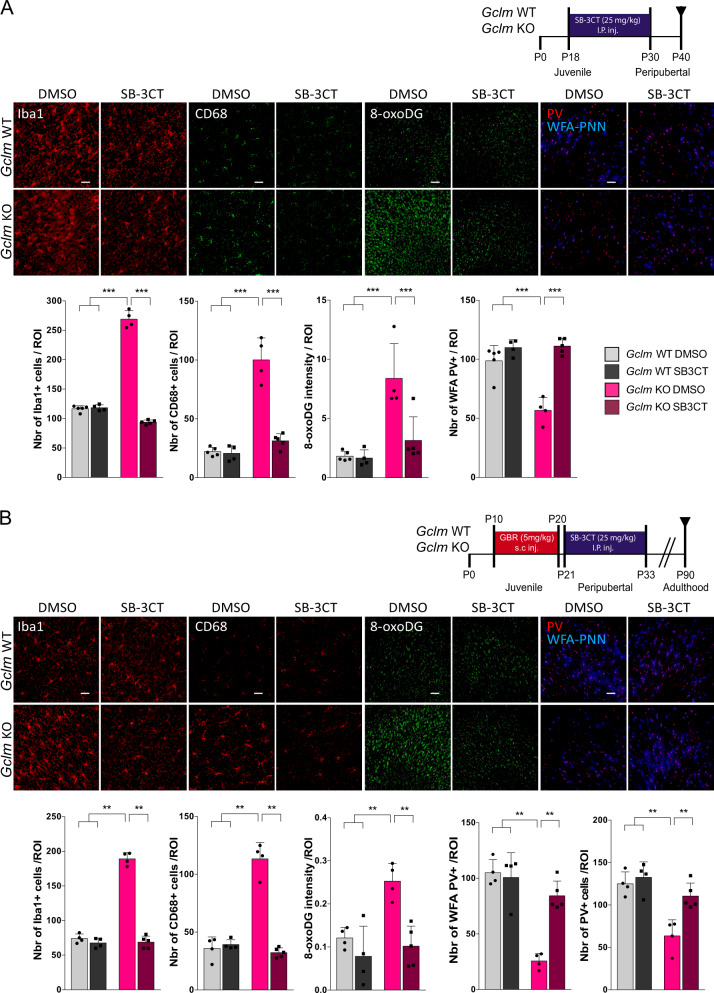
Inhibition of MMP9 by SB-3CT prevents OxS, microglia activation and the PNN decrease in the ACC of Gclm-KO at PND40, but also the long-term effect at PND90, when applied after an additional oxidative challenge. **a** Confocal images showing Iba1 (Scale bar: 30 μm), CD68 (Scale bar: 30 μm), 8-oxoDG (Scale bar: 50 μm) and parvalbumin (PV) staining enwrapped with the Wisteria floribunda lectin (WFA) (Scale bar: 50 μm) staining in Gclm-KO and WT mice after 4 injections of SB-3CT (25 mg/kg), each injection separated by 4 days, from PND18 until PND30 as shown in the schematic representation of the protocol and the corresponding quantification graph. **b** Confocal images showing Iba1 (Scale bar: 30 μm), CD68 (Scale bar: 30 μm), 8-oxoDG (Scale bar: 50 μm) and parvalbumin (PV) staining enwrapped with the Wisteria floribunda lectin (WFA) (Scale bar: 50 μm) and the corresponding quantification graph at PND90 after GBR (5 mg/kg) injection on PND10-20 followed by 4 injections of SB-3CT (25 mg/kg), each injection separated by 4 days, from PND21 until PND33, as shown in the schematic representation of the protocol. **a**, **b** Data are expressed as the mean ± s.e.d. (*n* = 5–6). ** *P* < 0.01; ***P < 0.001; analyzed by 2-way ANOVA followed by Tukey post hoc test. I.P.inj., intraperitoneal injection; s.c. inj., subcutaneous injection

We then investigated the impact of MMP9 on OxS and neuroinflammation propagation, followed by an investigation of its effect on the alteration of PNNs, the first components affected by OxS due to their role as a protective shield for PVIs [19], observed at PND40 in the *Gclm*-KO mice [18]. *Gclm*-KO and WT mice were injected IP with an SB-3CT (25 mg/kg) or control solution (DMSO/PEG-2000) four times during the PVI/PNN maturation sensitive period (PND18-30; 4 injections, each injection separated by 4 days) (Fig. 3a), and markers of mitochondrial DNA oxidation (8-oxoDG), microglia activation (Iba1 and CD68), NFkB activation, RAGE shedding, and MMP9 protein/activity, as well as PVIs/PNNs, were evaluated at PND40. As with the acute injection of SB-3CT, the chronic SB-3CT injection decreased RAGE shedding and MMP9 protein/activity in the *Gclm*-KO mice compared to the control treatment but had no effect in the WT (Fig. 2d, Suppl. Figure 4A–B). Interestingly, both 8-oxoDG intensity and microglia activation (Iba1 and CD68) were also normalized by SB-3CT treatment, suggesting that MMP9 inhibition could indeed prevent the pro-inflammatory and OxS processes (Fig. 3a). The effect of MMP9 inhibition on NFkB activation was also assessed through injection of AAV9-NRE-eGFP/ AAV9-CMV-mCherry into the lateral ventricle of PND20 *Gclm*-KO mice who were also treated with either SB-3CT or control from PND18 until PND30. Importantly, NFkB activation was lower in the SB-3CT-injected animals than in those injected with the control solution (Fig. 2i). Finally, the reduced PNN level in *Gclm*-KO mice at PND40 was restored by MMP9 inhibitor treatment (Fig. 3a).

### Long-term effect of MMP9 inhibition on PVIs and PNNs

We further investigated whether inhibition of MMP9 and the resulting RAGE shedding in an early developmental stage prevented the long-lasting effect of additional environmental insults on PVI/PNN expression as observed in adulthood [18]. Here, an additional oxidative challenge was induced by injecting GBR (5 mg/ml/kg), a dopamine reuptake inhibitor, to mimic the increase in dopamine release in the prefrontal cortex (PFCx) induced by environmental insults [55], leading to increased reactive oxygen species (ROS) through dopamine catabolism [56]. Indeed, this insult during the period of PVI maturation (PND10-20) led to a long-lasting PVI/PNN deficit at PND90 only in the OxS-vulnerable *Gclm*-KO mice.

To inhibit the effect of early GBR treatment on OxS, microglia and PVIs/PNNs, four injections of SB-3CT (25 mg/kg, PND21-34, 4 injections, each injection separated by 4 days) were given after GBR treatment, and the 8-oxoDG intensity and microglia activation were evaluated in adult mice (PND90). As expected, an increase in 8-oxoDG intensity and microglia activation after juvenile GBR treatment was observed in *Gclm-*KO mice compared to WT mice. SB-3CT treatment prevented this effect in the *Gclm-*KO mice but had no effect on WT mice (Fig. 3b). Additionally, the reduction in PVI+ and PNN+ PVI cells (PVI cells enwrapped in a PNN) in *Gclm-KO* mice after GBR treatment was normalized by the MMP9 inhibitor to levels close to those in WT mice (Fig. 3b).

Altogether, these results suggest that inhibition of MMP9 activation at the peripubertal stage can prevent the additional OxS-induced impaired maturation of PVIs and PNNs.

### Translational application: the sRAGE plasma level is increased in EP patients and predicts an I/E imbalance

To evaluate whether peripheral sRAGE could be an early mechanistically based marker of a central I/E imbalance reflecting inhibitory circuitry impairment, we investigated the relationship between peripheral sRAGE levels and medial PFCx (mPFCx) GABA levels in a large cohort of young patients (*n* = 111 for plasma, 33 for magnetic resonance spectroscopy (MRS)), as compared to control subjects (*n* = 68 for plasma, 39 for MRS) (Table 1). Notably, tested EP SZ patients were at an early stage of the disease (age: 24.4 ± 4, and illness duration: 2 ± 1.8, mean ± SD, in years for both). In particular, we tested whether differences in sRAGE were more prominent in high-risk GAG GCL genotype subjects (patients and controls) who, similar to *Gclm*-KO mice, display decreased mPFCx GSH levels [37] and increased vulnerability to OxS [9]. Here, we examined the correlation of plasma sRAGE levels, measured by ELISA, with mPFCx GABA levels and the GABA/glutamate ratio, assessed by MRS [37].

**Table 1:** Demographic table for early psychosis patients and healthy controls enrolled in the study

	Controls (*n* = 68/*39*)	Patients (*n* = 111/*33*)	*p*-value
Age (years, mean ± s.d.)	25.3 ± 4; *25.08* ± *4.7*	24.4 ± 4; *23.9* ± *4.7*	0.133^a^ *0.27*^a^
Gender	43 men; 25 women	84 men; 27 women	0.09^b^
	20 men; *19 women*	*24 men; 9 women*	*0.08*^b^
Cigarette users/non-users	8/54; *6*/*29*	41/39; *15/18*	< 0.001^b^; *0.017*^b^
BMI (kg/m^2^, means ± s.d.)	23.03 ± 2.9; *22.57* ± *2.5*	24.1 ± 3.6; *23.5* ± *3.3*	0.06^a^; *0.19*^a^
GAG-GCL polymorphism (LR/HR)	47/21; *25/14*	77/34; *21/12*	1^b^; *1*^b^
Illness duration (years, mean ± s.d.)	NA	2 ± 1.8; *2* ± *1.7*	NA
CPZ equivalent (mg, mean ± s.d.)	NA	337.9 ± 276.4; *385.1* ± *316.5*	NA

*CPZ equivalents* chlorpromazine equivalents, *n.s.* not significant (*P* > 0.05), *LR* low risk, *HR* high risk

In italics: subjects with a magnetic resonance spectroscopy (MRS) scan

^a^t-test

^b^Fisher exact test

**Fig. 4:**
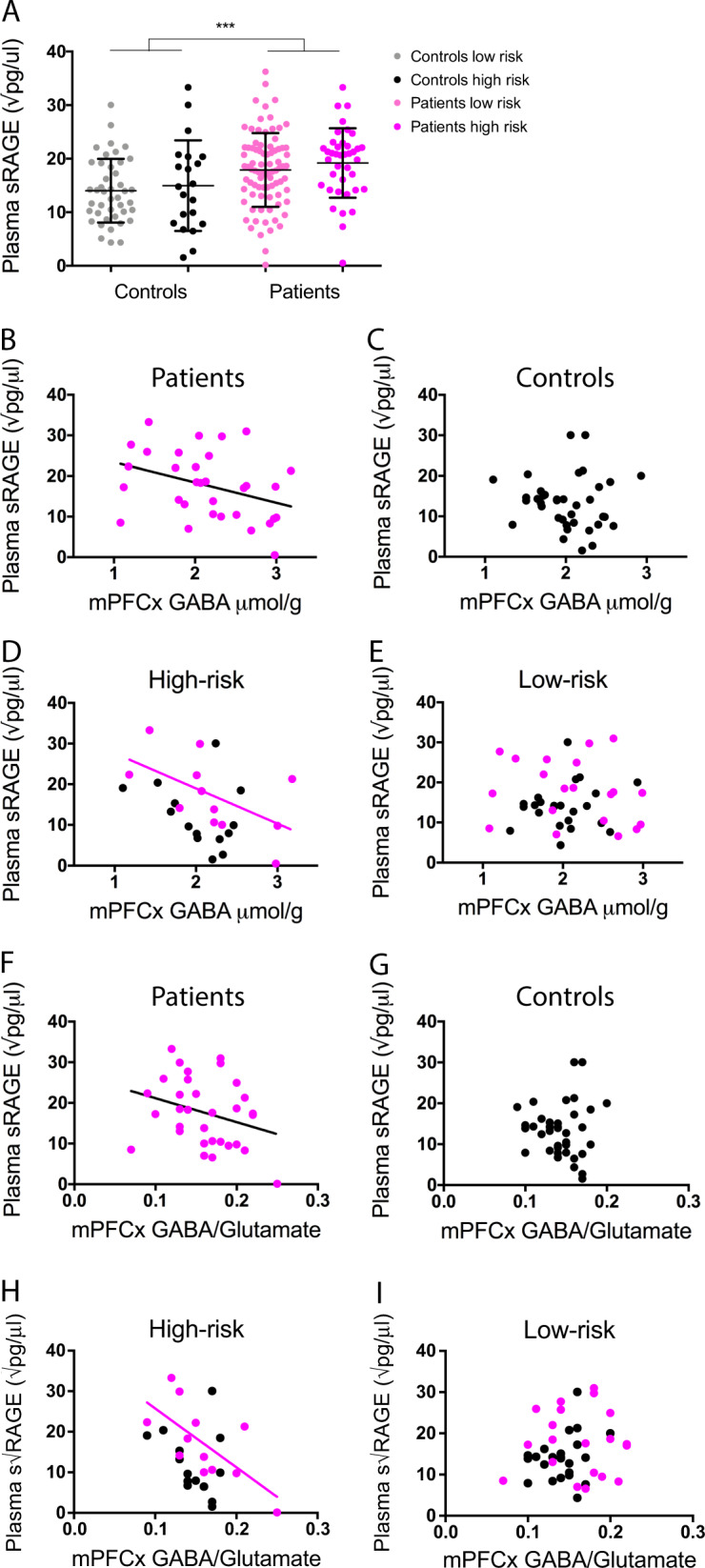
Plasma sRAGE level is higher in early psychosis patients than in healthy controls and is negatively correlated to PFCx GABA. **a** Graph showing the increased sRAGE level in plasma of patients (*N* = 111) compared to that in controls (*N* = 68; *p* = 0.001). **b** Plasma sRAGE is negatively correlated to mPFCx GABA in patients (*N* = 33; *r* = −0.36, *p* = 0.039) **c** but not in controls (*N* = 39; *r* = −0.07, *p* = 0.649). **d** Plasma sRAGE is negatively correlated to mPFCx GABA in the high-risk genotype (*N* = 26), an effect that is driven by the patients (*N* = 12; *r* = −0.32, *p* = 0.05), **e** but not in the low-risk genotype (*N* = 46; *r* = −0.05, *p* = 0.72). **f** Plasma sRAGE also had a high tendency to negatively correlate with the ratio of mPFCx GABA/mPFCx glutamate in patients (*N* = 33; *r* = −0.29, *p* = 0.098) **g** but not in controls (*N* = 39; *r* = −0.02, *p* = 0.88). **h** This negative correlation is significant in the high-risk genotype (*N* = 26), driven by the patients (*N* = 12; *r* = −0.46, *p* = 0.014), **i** but not in the low-risk genotype (*N* = 46; *r* = 0.14, *p* = 0.367). ***P < 0.001

sRAGE levels were higher in patients than in controls (*p* = 0.001) (Fig. 4a). Using a linear regression model, no effects of gender (*p* = 0.5), age (*p* = 0.49), age at first psychosis (*p* = 0.26), medication (*p* = 0.73), GAG polymorphism genotype (*p* = 0.18) or life style factors (cigarette smoking (*p* = 0.87) and body mass index (BMI) (*p* = 0.66)) were found. However, subject status (control vs. patient), the level of mPFCx GABA and the GABA/glutamate ratio exhibited a significant effect on the sRAGE level, with an interaction of the GAG polymorphism with the GABA levels and with the GABA/glutamate ratio. Interestingly, the mPFCx GABA level was negatively correlated with sRAGE in patients but not in controls; namely, high levels of peripheral sRAGE (due to increased RAGE shedding) were associated with low mPFCx GABA levels (Fig. 4b, c, *r* = −0.36; *p* = 0.039). Moreover, GABA levels were also negatively correlated with sRAGE in high-risk genotype subjects, an effect mainly driven by the patients (Fig. 4d, *r* = −0.32, *p* = 0.05). In contrast, no such correlations were observed in the low-risk genotype subjects (Fig. 4e). This result was also corroborated by the GABA/glutamate ratio, which was negatively correlated with sRAGE levels in the OxS-vulnerable, high-risk genotypes (Fig. 4h, *r* = −0.46, *p* = 0.014), an effect mainly driven by the patients (Fig. 4f–i).

In line with the results in the animal model (*Gclm*-KO mice), a higher level of RAGE shedding is associated with a reduction in brain GABA and the GABA/glutamate ratio in EP SZ patients, especially in subjects with genetic vulnerability to OxS.

## Discussion

**Fig. 5:**
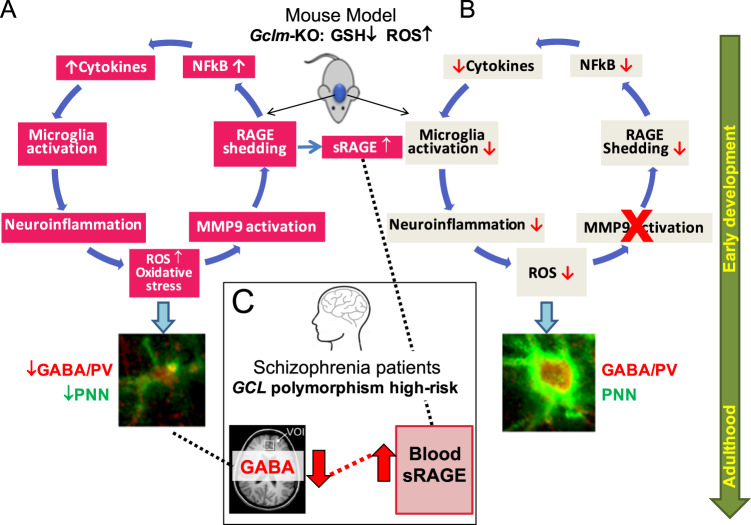
Involvement of MMP9/RAGE shedding in the interaction between OxS and neuroinflammatory processes potentiating one another in a damaging positive feedforward mechanism that lead to PVI maturation impairments. **a** In the preclinical model of redox dysregulation, activation of MMP9 by ROS via its cysteine switch induces RAGE shedding in neurons, with translocation of its intracellular domain to the nucleus and secretion of a soluble form (sRAGE). NF-kB activation, potentially mediated by Intra-RAGE translocation to the nucleus, induces pro-inflammatory cytokines and MMP9 secretion, microglia and astrocyte activation and further ROS production. This self-maintaining mechanism, occurring early during development, affects PVI maturation as shown by decreased PV expression and PNN formation which last until adulthood. **b** Inhibition of MMP9 (cross in red) prevents all of these processes (downwards red arrows) and allows normal PVI maturation. **c** In parallel to the preclinical model, RAGE shedding, producing sRAGE, is increased in early psychosis patients and predicts low mPFCx GABA level in patients bearing the high-risk genotype of the GAG polymorphism in GCL gene

In a genetic mouse model of GSH deficit, increased OxS and consequently neuroinflammatory state affected PVI/PNN maturation, causing long-term deficits, especially after an additional oxidative insult during early postnatal development. Our results suggest a feedforward potentiation loop between OxS and neuroinflammation involving the following steps: activation of MMP9 by OxS, leading to RAGE shedding, followed by NFkB activation, secretion of pro-inflammatory cytokines, microglia activation and further ROS production and OxS during juvenile postnatal development (Fig. 5a).

MMP9 inhibition at early developmental stages prevented the PVI/PNN deficit in adulthood. Notably, this experiment was the first use of this inhibitor in the context of psychiatric disease and, more specifically, in the restoration of the PVI/PNN impairment in vivo. In our model, the neuroinflammation-induced by OxS was mediated by MMP9, as the MMP9 inhibitor prevented it. Therefore, the MMP9/RAGE pathway appears to play an important role in maintaining the feedforward loop between OxS and neuroinflammation. Although MMP9 may induce neuroinflammation through microglia activation [57], cytokine release and extracellular matrix degradation [58], the precise mechanism by which MMP9 activate NF-kB has not been demonstrated. Therefore, NF-kB activation may involve RAGE shedding. The classical RAGE activation pathway consists of activation of Full-RAGE by its ligands, such as S100B, which are secreted by astrocytes, leading to NF-kB or NADPH oxidase (NOX) activation [50, 59–61]. However, the role of RAGE shedding and more specifically the Intra-RAGE is poorly understood. Although sRAGE has been detected in the lung and biofluids of animal models and patients with different inflammatory diseases [44], this is the first time, to the best of our knowledge, that RAGE shedding has been visualized by IH in the brain in vivo. Moreover, the in vivo demonstration of the translocation of Intra-RAGE to the nucleus is also unique. Indeed, only two in vitro studies have suggested that this intranuclear domain could activate apoptosis [62] or cellular migration [63]. The potential activation of NFkB by Intra-RAGE, which has never been previously described, may be the missing connection linking OxS and neuroinflammation.

While the propagation of neuroinflammation and OxS by the activation of the MMP9/RAGE pathway affects PVI/PNN maturation in our model, MMP9 may also directly affect PVIs by inducing the degradation of their PNNs, the formation of which is an important maturation step [64]. MMP9 is known to degrade some proteins and receptors of the extracellular matrix and has recently been suggested to also degrade chondroitin sulfate proteoglycans (CSPGs), a main component of PNNs [65]. Moreover, MMP9 may induce PNN degradation through the activation of other MMPs. To date, CSPG degradation primarily involves ADAMTS4 and ADAMTS5 [66]. However, a recent study revealed the expression of some proteases specifically in PVIs, suggesting their potential involvement in PNN degradation [67]. We, therefore, propose that OxS-mediated activation of MMP9 during the early phase of PNN maturation may lead to RAGE shedding but may also activate PVI-specific MMPs that degrade PNNs.

PVIs and PNNs are sensitive to OxS and neuroinflammation, specifically during their maturation period as shown in a large number of SZ animal models carrying various genetic and environmental risks [13]. Intriguingly, these early deficits are maintained through life. Our results provide evidence of MMP9/RAGE mechanism occurring early during brain development and inducing a long-lasting effect on PVI/PNN integrity in *Gclm*-KO mice [18] by perpetuating the pathological process. Importantly, the MMP9 inhibitor is still effective after the additional OxS, when given during PVI maturation period. The peripubertal stage represents a key period of PFCx development in which PVI maturation ends, synapses are pruned, and the I/E synaptic balance is established [68–70]. Since microglia are involved in synaptic pruning [42, 71, 72], increased microglia activation at the peripubertal stage may affect synapse maturation, resulting in an increase in the number of immature spines. In addition to microglia, MMP9 has been also shown to be involved in synapses remodeling [73]. Interestingly, adolescents diagnosed with SZ show a drastic decline in gray matter volume throughout life [74], compatible with greater synaptic elimination [75], which may underlie the cognitive deficits observed in patients.

This MMP9/RAGE mechanism is a primary interest as some evidence implicates MMP9 and RAGE in SZ pathophysiology. For instance, patient serum contains higher levels of AGEs and S100B than serum from healthy controls [76, 77]. Different studies have reported conflicting results on the levels of sRAGE in serum, showing increased [30] or decreased sRAGE [77, 78] in patient serum. Of note, our study was the first to measure sRAGE in young patients at an early stage of the disease (mean age of 24.4 ± 4 years and illness duration of 2 ± 1.8 years), while Takeda et al. and Emanuele et al. investigated aged patients at a more chronic state. Interestingly, the aged SZ patients had lower levels of sRAGE than healthy controls, suggesting that increased sRAGE is linked to the early phase of the disease, in line with the *Gclm-*KO mice. MMP9 in patients was found to be increased compared to healthy controls and was correlated with OxS markers in the serum of patients [27, 79]. At the genetic level, a polymorphism in the genes for RAGE and MMP9 was associated with psychotic personality trait in a normal population and SZ patients [28, 80].

In our study, the levels of sRAGE were increased in patients at the early stage of the disease compared to those in healthy controls and were associated with low mPFCx GABA levels in patients with a genetic susceptibility to OxS. This link between sRAGE and the central inhibitory system is in line with our preclinical model in which OxS-induced MMP9 activation and increased RAGE shedding lead to PVI deficits (Fig. 5a and c). Moreover, the association between plasma sRAGE and brain GABA found in the high-risk GAG GCL genotype (low GSH) further highlights the role of OxS in this association. Similar to the genetic preclinical model of redox dysregulation, individuals with a genetic vulnerability to OxS showed increased sRAGE associated with a lower GABA level compared to those without the same vulnerability. Whether the sRAGE detected in the plasma is secreted by peripheral immune cells or is released by the cerebrospinal fluid remains to be determined. In any case, sRAGE may be a potential mechanistically based biomarker that may be indicative of an inhibitory transmission deficit in patients.

Based on our results, the MMP9/RAGE pathway appears to be a very promising target for novel drug development in psychiatry. In our study, an MMP9 inhibitor was used as a proof-of-concept to interfere with the key mechanisms mediating the dysregulated redox-inflammation interaction to prevent long-term PVI deficits. As MMP9 has widespread functions both in the brain and periphery [51], the treatment of patients with MMP9 inhibitors is not conceivable. Therefore, the development of negative and positive allosteric modulators of this pathway is warranted. Importantly, the ability of the MMP9 inhibitor to prevent PVI impairments when given after the additional OxS is of particular clinical interest, as, in analogy, an intervention following the second environmental hit might have a therapeutic effect in patients.

Early diagnosis and intervention in psychosis and SZ, an important focus of psychiatry [81], is hampered by the lack of valid biomarkers. Previous reports on peripheral cytokine anomalies in SZ gave mixed results, and the peripheral levels do not necessarily correlate with their central levels [82–84]. Our translational study allowed for the establishment of a mechanistically related biomarker profile based on dysregulation of the redox-inflammation interaction, paving the way towards early detection, stratification of patients, and the ability to monitor potential drug effects.

## Supplementary information

Supplementary Figure Legends

Supplementary Figure 1

Supplementary Figure 2

Supplementary Figure 3

Supplementary Figure 4

Supplementary Materials and Methods
