# Supplementary material for: MMP9/RAGE pathway overactivation mediates redox dysregulation and neuroinflammation, leading to inhibitory/excitatory imbalance: a reverse translation study in schizophrenia patients
Source: Mol Psychiatry. 2019 Mar 25;25(11):2889–904. doi: 10.1038/s41380-019-0393-5 (PMC7577857; doi:10.1038/s41380-019-0393-5)
Supplement: Supplementary file 1 — Supplementary Figure Legends [file 41380_2019_393_MOESM1_ESM.docx]

**Supplementary Figure Legends**

**Supplementary Figure 1.** Astrocyte activation is increased in the ACC of Gclm-KO compared to that in the ACC of WT mice at PND40. No microglia activation in the somatosensory cortex of Gclm-KO mice at PND40. (A) Schematic representation of the ACC, followed by confocal images (Scale bar: 30 μm) showing S100B staining and the quantification graph of the cell number at PND40. (B) Schematic representation of somatosensory cortex, where Iba1 and CD11b staining were visualized by confocal images (Scale bar: 30 μm) and quantified in Gclm-KO and WT mice at PND40. (A-B) Data are expressed as the mean ± s.e.d. (n = 5-6). ** P < 0.01; analyzed by Student’s t test.

**Supplementary Figure 2.** RAGE shedding and MMP9 protein are increased in the ACC of Gclm-KO compared to that in the ACC of WT mice at an early stage of development. (A) Schematic representation of MMP9 activation by oxidative stress via its cysteine switch. (B) Confocal images (Scale bar: 30 μm) of RAGE shedding with the corresponding quantification graph (Intra-RAGE/Extra-RAGE) for PND20, PND40 and PND90. (C) Confocal images (Scale bar: 30 μm) showing MMP9 staining and the corresponding quantification graphs for PND20, PND40 and PND90. (B-C) Data are expressed as the mean ± s.e.d. (n = 5-6). Analyzed by 2-way ANOVA with 2 factors (genotype and age) followed by Tukey post hoc test. For genotype effect: **** P < 0.0001, *** P < 0.001, ** P < 0.01; For age effect: &&& P < 0.001, && P < 0.01, & P < 0.05.

**Supplementary Figure 3.** Methodological validation of the MMP2/9 inhibitor SB-3CT for peripheral injections. (A) Schematic representation of the SB-3CT intracortical injection site and protocol. PND40 Gclm-KO mice were sacrificed 5 min after SB-3CT injection at 3 different doses (50 µM, 27 µM, 9 µM), and brains were dissected to obtain the tissue containing the injection site and the tissue anterior to the injection site. The ipsilateral and contralateral Cortex (Cx) were separated in each slice containing the injection site and anterior to the injection site. MMP9 activity was measured by gelatin zymography and is represented in the graph. (B) Schematic representation of the SB-3CT intraperitoneal injection protocol. PND10 Gclm-KO mice were sacrificed 2 h or 4 h after injection, and brains were then dissected to obtain 3 slices, an anterior, middle and posterior slice of the Cx, for the detection of MMP9 activity, represented in the graph. (A-B) Data are expressed as the mean ± s.e.d. (n = 2-5). *** P < 0.001, ** P < 0.01; analyzed by 2-way ANOVA and Tukey post hoc test. SB, SB-3CT; Vcl, vehicle; I.P.inj., intraperitoneal injection.

**Supplementary Figure 4.** Inhibition of MMP9 by SB-3CT prevents RAGE shedding and decreases the MMP9 protein level in the ACC of Gclm-KO mice compared to that in the ACC of WT mice at PND40. (A) Confocal images (Scale bar: 30 μm) showing RAGE shedding in Gclm-KO and WT mice after 4 injections of SB-3CT (25 mg/kg), each injection separated by 4 days, from PND18 until PND30 as shown in the schematic representation of the protocol. The graph represents the Intra-RAGE/Extra-RAGE ratio. (B) Confocal images (Scale bar: 30 μm) showing the MMP9 protein level at PND40 after SB-3CT injections and the corresponding quantification graph. (A-B) Data are expressed as the mean ± s.e.d. (n = 5-8). *** P < 0.001; analyzed by 2-way ANOVA and Tukey post hoc test. I.P.inj., intraperitoneal injection.
