# Supplementary figures and images for: MMP9/RAGE pathway overactivation mediates redox dysregulation and neuroinflammation, leading to inhibitory/excitatory imbalance: a reverse translation study in schizophrenia patients
Source: Mol Psychiatry. 2019 Mar 25;25(11):2889–904. doi: 10.1038/s41380-019-0393-5 (PMC7577857; doi:10.1038/s41380-019-0393-5)

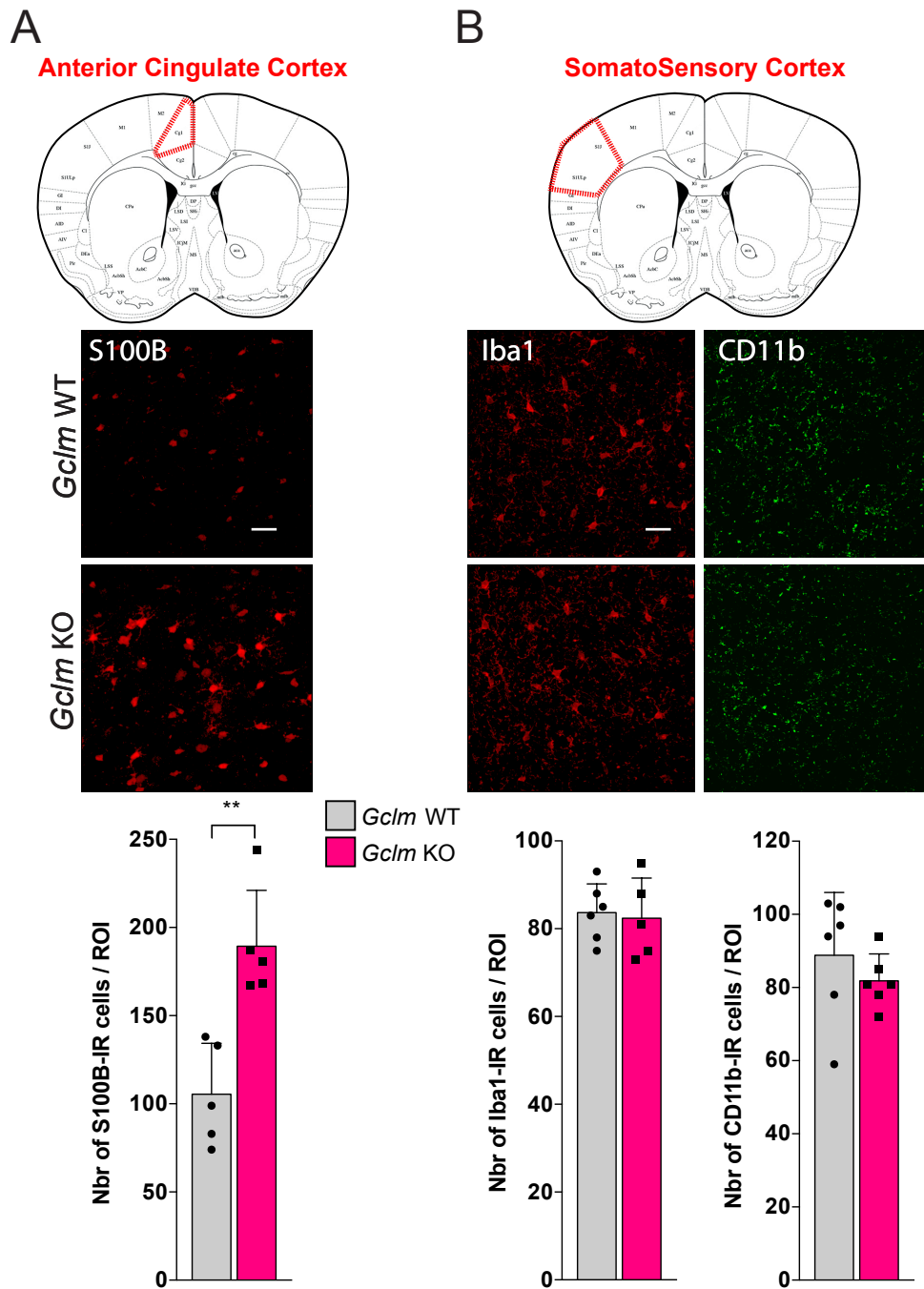

Supplementary Figure 1

Supplement: Supplementary file 2 — Supplementary Figure 1 [file 41380_2019_393_MOESM2_ESM.pdf]

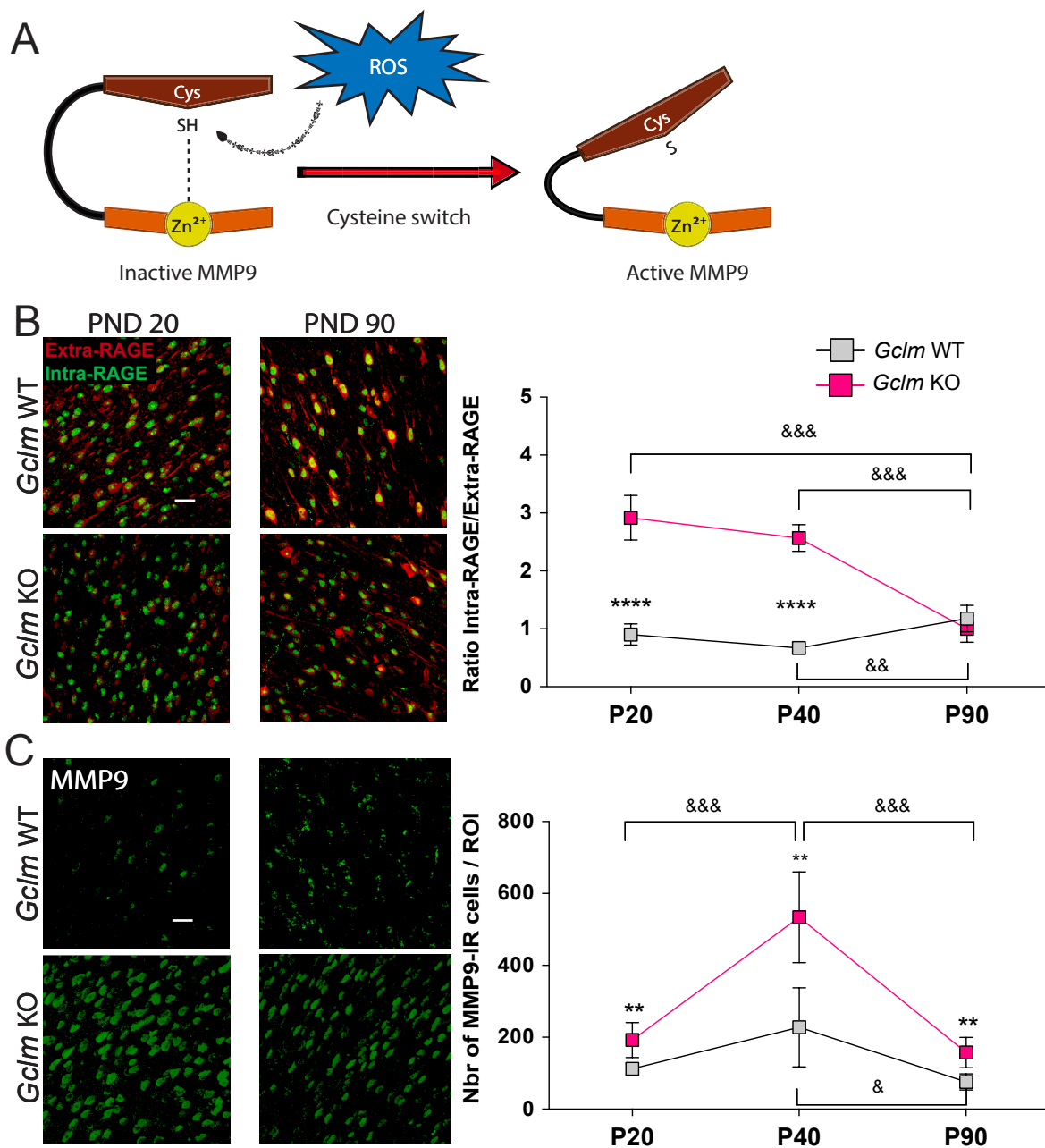

Supplementary Figure 2

Supplement: Supplementary file 3 — Supplementary Figure 2 [file 41380_2019_393_MOESM3_ESM.pdf]

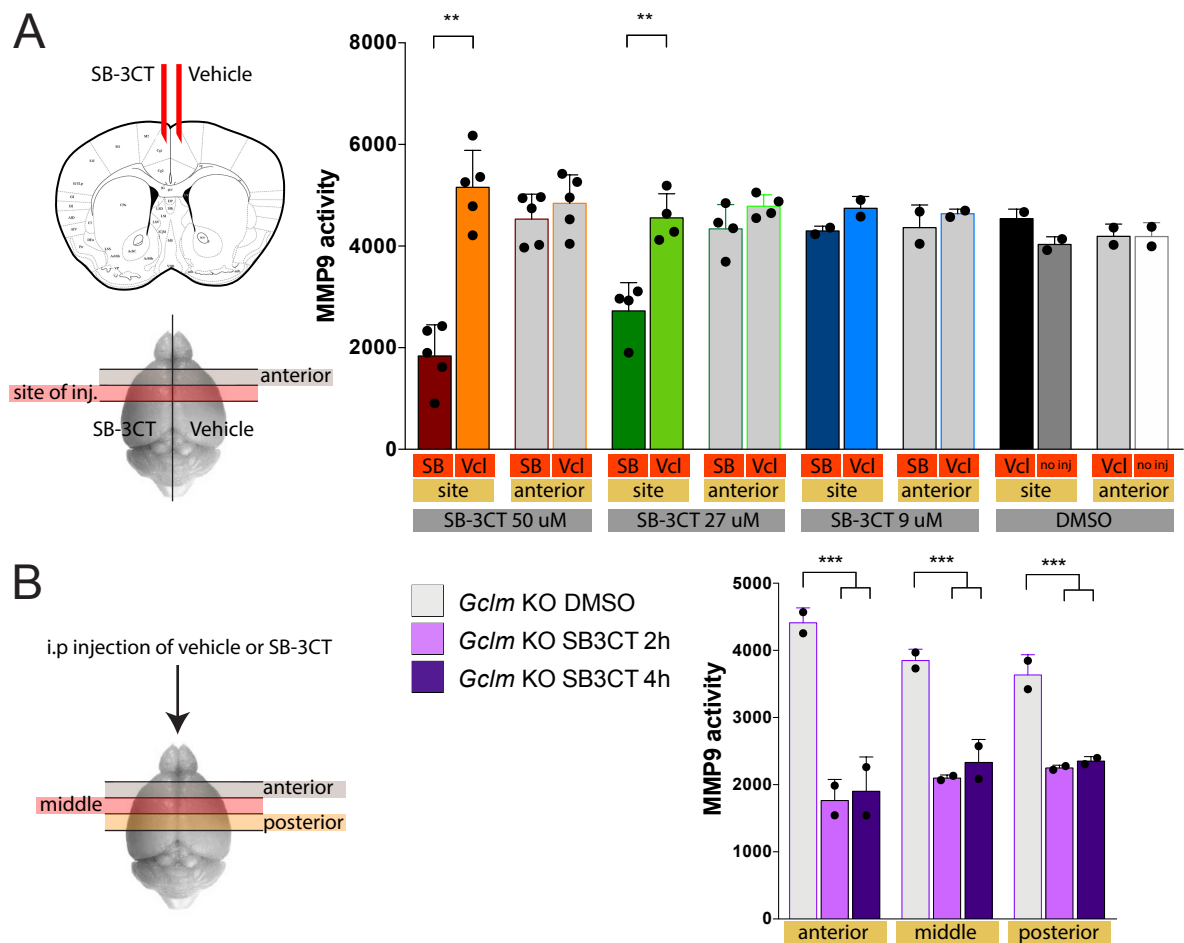

Supplementary Figure 3

Supplement: Supplementary file 4 — Supplementary Figure 3 [file 41380_2019_393_MOESM4_ESM.pdf]

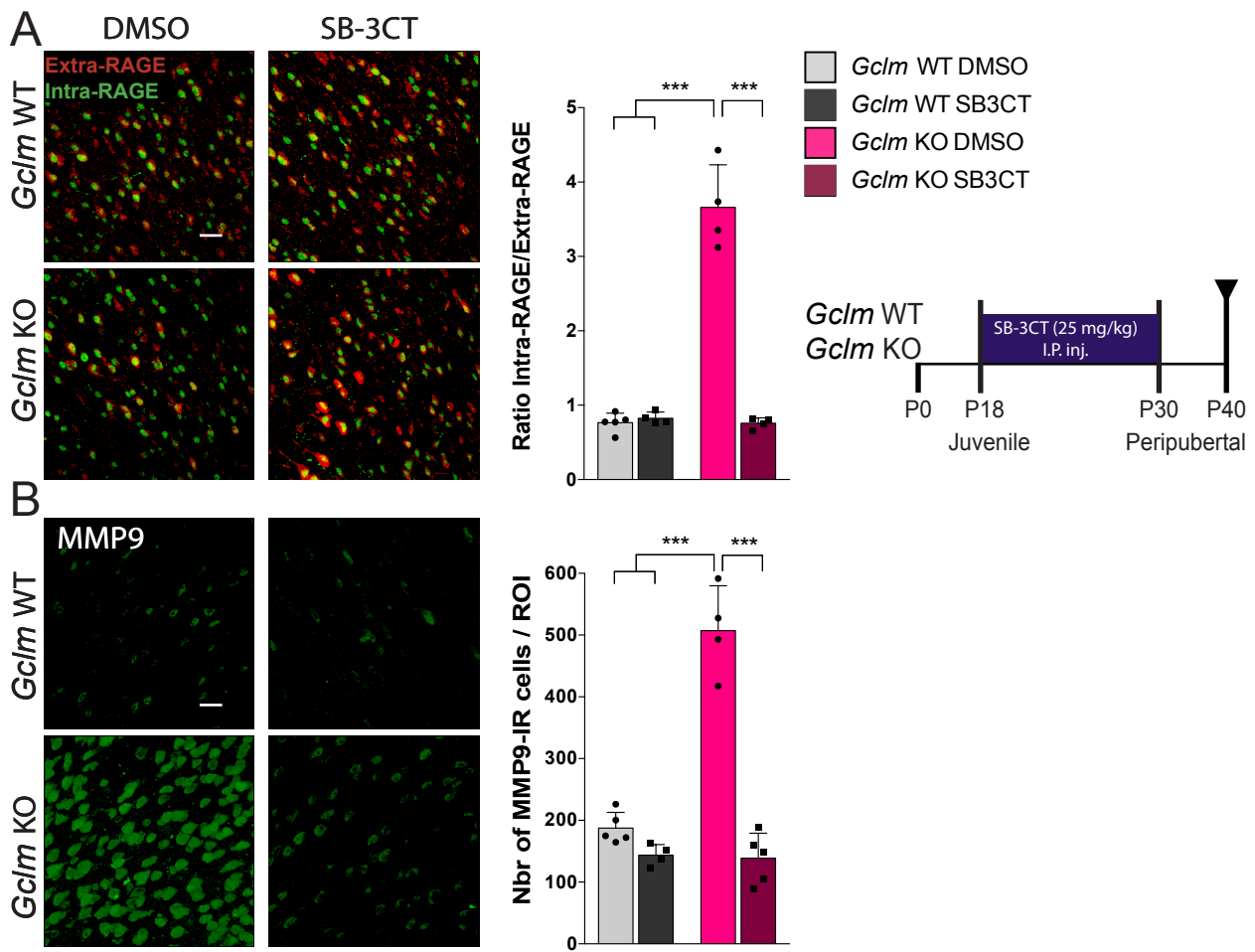

Supplementary Figure 4

Supplement: Supplementary file 5 — Supplementary Figure 4 [file 41380_2019_393_MOESM5_ESM.pdf]
