# Supplementary material for: MMP9/RAGE pathway overactivation mediates redox dysregulation and neuroinflammation, leading to inhibitory/excitatory imbalance: a reverse translation study in schizophrenia patients
Source: Mol Psychiatry. 2019 Mar 25;25(11):2889–904. doi: 10.1038/s41380-019-0393-5 (PMC7577857; doi:10.1038/s41380-019-0393-5)
Supplement: Supplementary file 6 — Supplementary Materials and Methods [file 41380_2019_393_MOESM6_ESM.docx]

**Supplementary Materials and Methods**

**Mice**

The *Gclm-K*O mice were previously generated by Yang et al.^37^. and provided by T. Dalton (University of Cincinnati). These mice were backcrossed with C57BL/6J mice and maintained as described previously^33^. Experiments were done on males only and were first approved by the Swiss cantonal veterinary office.

**Subject recruitment**

All patients were recruited from the Treatment and Early Intervention in Psychosis Program (TIPP)^38^, a 3-year specialized early psychosis program in the department of psychiatry at Lausanne University Hospital, Switzerland. Eligibility criteria for the program were: (I) age between 18 and 35; (II) living in the catchment area (Lausanne and surroundings); (III) meeting threshold criteria for psychosis, as defined by the “Psychosis threshold” subscale of the Comprehensive Assessment of At Risk Mental States (CAARMS)^85^ Scale. Patients were not eligible and referred to other treatment programs if they had been taking antipsychotic medication for more than 6 months, had psychosis related to intoxication or organic brain disease, or had an IQ below 70. Patients with an illness duration of more than 5 years were excluded.

Healthy controls were recruited from similar geographic and sociodemographic areas through advertisement and assessed by the Diagnostic Interview for Genetic Studies^86^. Controls were excluded from the study if they met one of the following criteria: had a major mood, psychotic, or substance-use disorder or had a first-degree relative with a psychotic disorder. Furthermore, neurological disorders and severe head trauma were exclusion criteria for all participants (i.e., patients and controls). Healthy controls were matched for sex, age and cigarette smoking (i.e., user or non-user). BMI (kg/m^2^) was calculated for all participants. For patients, symptomatic severity was assessed by a trained psychologist using the Positive and Negative Syndrome Scale (PANSS)^87^. Antipsychotic doses at the time of the study were converted to chlorpromazine equivalents (CPZ equivalents in mg) for each patient^88^. At the time of blood sampling, 101 patients out of 111 were receiving antipsychotic medication (c.f. Table 1). All assessments (MRS, plasma and clinical) were performed at the same time point (plasma measurement of sRAGE was done on 68 healthy subjects and 111 patients, while MRS analysis was conducted on 39 healthy subjects and 33 patients). The GAG trinucleotide-repeat polymorphism in *Gclc* gene was genotyped as previously described^9^ and assigned into *Gclc* high-risk or *Gclc* low-risk genotype based on the number of GAG repeats as defined in Gysin et al.^9^. As no SNP haplotype has been found to be a perfect surrogate for any of the GAG repeat lengths in *Gclc* gene, they cannot be derived from available SNP data from genome-wide association studies (GWAS) and a standard methodology should be used to correctly genotype these alleles^89^. Informed written consent in accordance with our institutional guidelines (protocol approved by the Ethic Committee of Lausanne University) was obtained for all subjects.

**Intracortical injection of SB-3CT and brain tissue dissection**

Since this MMP9 inhibitor has mainly been used in ischemic models when the BBB is known to be leaky, some preliminary experiments were conducted to test the efficacy of SB-3CT and to determine the right dose. First, this inhibitor was intracortically injected into the ACC of PND40 *Gclm-K*O mice at different doses, and the brain tissue was dissected to measure for MMP9 activity using gelatin zymography. Intracortical injection into the ACC was done at the following coordinates: AP = 0.2, L = 0.28, and V = 0.2, and SB-3CT was injected at 50 µM, 27 µM and 9 µM, diluted in 25% DMSO, 65% PEG-2000 and 10% water into the right hemisphere, while the vehicle solution was injected into the left hemisphere. The effect of the injection itself on MMP9 activity was controlled by comparing the effect of vehicle solution injection on one side to that of no injection on the other.

After SB-3CT injection, brain tissue was dissected after 5 min using a brain matrix. One brain slice of 1-mm thickness was cut around the injection site (bregma ~ 0 - 1 mm), which contained the ACC, and then the Cx was separated into left and right hemispheres (Suppl Fig 3A). Another anterior slice of 1-mm thickness was dissected (bregma ~ 1 - 2 mm) to control for SB-3CT diffusion, and the left and right hemispheres were separated (Suppl Fig 3A).

The capacity of SB-3CT to reach the brain was evaluated by IP injecting PND10 mice because of the leaky state of the BBB at this age. Then, MMP9 activity was measured in different brain regions 2 h and 4 h after the SB-3CT (50 µM) injection in PND10 animals. Anterior (bregma ~ 1 - 2 mm), middle (bregma ~ 0 - 1 mm) and posterior (bregma ~ - 1 - 0 mm) slices were dissected, and the Cx was isolated for MMP9 activity measurements (Suppl Fig 3B).

**Adeno-associated viral vectors**

The NFkB-inducible AAV vector has been previsouly described^50^. Briefly, eight copies of the JC virus NFkB-responsive sequence were fused to a minimal cytomegalovirus (CMV) promoter, generating the pSC-NF-d1-eGFP AAV vector plasmid. To produce recombinant AAV9-2YF-NRE-eGFP virus, pSC-NF-d1-eGFP was co-transfected with the pAd Delta F6 plasmid (a kind gift from the Pen Vector Core, University of Pennsylvania) and pAAV2/9-2YF packaging plasmid (a kind gift from Dr D.Dalkara^90^) into HEK-293T cells and the viral stock was purified by the iodixanol method^51^. HEK293 cell line were tested negatively for mycoplasma contamination and authentication has been performed by supplier manufacture. The titer of the resulting viral vector, evaluated by quantitative PCR using primers located in the SV40 polyA sequence, was 4.5 x 10^13^ vg/mL.

The AAV9.CB7.CI.mCherry.WPRE.rBG (hereafter AAV9-CBA-mCherry) vector was purchased from the Penn Vector Core (University of Pennsylvania; cat n° V3874TI-R; titer: 1.49 x 10^13^ vg/mL, AAV9.CB7.CI.mCherry.WPRE.rBG).

The AAV9-2YF-NRE-eGFP virus was mixed with AAV9-CBA-mCherry at a final titer of 6.5 x 10^12^ and 1.15 x 10^11^ vg/mL, respectively. Surgery was done on PND20 *Gclm*-KO and WT mice with coordinates AP = 0.2, L = 0.8, and V = 2 for 2 ul injection into the right lateral ventricle. We based our protocol on previous studies^91^ that showed a wide diffusion of AAV into the Cx when injected into the ventricle, preventing tissue damage to the region of interest. After surgery, 2 to 3 mice were kept in a cage and left untouched until PND40, when they were sacrificed by intracardiac perfusion. In the chronic SB-3CT injection experiment, *Gclm*-KO mice received the first IP injection of the inhibitor two days before surgery (PND18) and then 3 others, separated by 4 days each.

**Immunohistochemistry**

Animals were sacrificed at PND40 or PND90 by intracardial perfusion of filtered 4% paraformaldehyde in PBS, pH 7.4. Coronal slices 40 µm thick were then cut with a microtome (Microm HM440E).

To identify RAGE shedding by IH, we used an antibody against the extracellular domain (Extra-RAGE) and another that recognizes the intracellular domain of RAGE (Intra-RAGE). The IH protocol was adapted from the classic method in order to avoid background and interference between both antibodies. Briefly, all IH steps were first performed for the Extra-RAGE antibody without the introduction of a permeabilization step, as the target is extracellular. Then, the slices were permeabilized to complete the IH for the Intra-RAGE antibody. These steps were first tested with different permeabilization and antibody concentration conditions (data not shown) to test the specificity of the antibodies. The antibody against Extra-RAGE (mouse/rat anti-RAGE, MAB1179, R&D systems, 1/3000) was used in 15% bovine serum albumin (BSA) blocking solution, followed by the antibody against Intra-RAGE (rabbit polyclonal to RAGE, ab3611, Abcam, 1/1500) in 2% normal goat serum (NGS) and 0.3% Triton X-100 blocking solution.

For the IH quantification of other proteins, the following antibodies were used: anti-NeuN (Millipore, MAB377, 1/2500), anti-Iba1 (Abcam, ab5076, 1/1000), anti-CD68 (Abcam, ab53444, 1/1500), anti-CD11b (Bio-Rad, MCA74GA, 1/1000), anti-S100B (Sigma, S2657, 1/1000), anti-MMP9 (Santa Cruz, sc-10737, 1/500), anti-8-oxoDG (Trevigen, 4354-MC-050, 1/350), anti-PV (Swant, PV 25, 1/50000), and anti-WFA (PNN) (Sigma, L1516, 1/50000).

**Confocal analysis and image analysis**

Images for protein quantification were obtained with a Zeiss LSM 780 Quasar confocal microscope equipped with 40x and 20x objectives. For PVI/PNN/8-oxoDG staining, the ACC brain region was scanned with the 20x objective with the area of interest being contained within one image. For other stainings, the 40x objective was used, covering the ACC area with 4 images. Two to three brain slices were analyzed per animal, and five to eight animals were used per experiment. A z-stack scan of 10 to 20 images per section, depending on the staining, was performed.

Processing and quantification of the images were performed with Imaris 7.4 software. Immunoreactive cells were counted using the Imaris spot tool, with a defined size threshold depending on the staining. Moreover, the labeling intensity was quantified for some stainings (8-oxoDG).

**ELISA for IL-6, IL-1β, and TNF**α

For the cytokine measurements, animals were quickly sacrificed by decapitation without anesthesia, and the PFCx was dissected using a brain matrix (bregma ~ 1.18 – 1.7 mm). The brain tissue was lysed with a lysis buffer composed of 100 mM TrisHCl pH 7.4, 150 mM NaCl, 2 mM EDTA, 1% Triton X-100, 1 mM PMSF, and 1 mM Na orthovanadate. IL-6 (eBioscience, 88-7064), IL-1β (eBioscience, 88-7013) and TNFα (eBioscience, 88-7324) ELISA kits were used as specified in the provided instructions.

**Tissue fractioning and Western blot**

For tissue fractioning, animals were quickly sacrificed by decapitation without anesthesia, and the ACC was dissected using a brain matrix (bregma ~ 0 – 1 mm). Brain tissue was first lysed with a Hepes buffer supplemented with 0.32 mM sucrose. After centrifugation at 2,000 x g for 10 min at 4°C, supernatant containing cytoplasmic proteins was collected. The pellet was then lysed with a lysis buffer containing 1% SDS, 2 mM EDTA, 50 mM TrisHCl, 150 mM NaCl, 1 mM PMSK, 1 mM Na orthovanadate, 1 mM Na fluoride and 5 mM DTT and centrifuged at 10,000 x g for 10 min at 4°C. The supernatant, containing the nuclear proteins, was collected. Cytoplasmic and nuclear proteins were then loaded onto a 12% SDS gel, and the specificity of the compartment isolation was tested by blotting anti-alpha-tubuline (mouse monoclonal, TU-02, Santa Cruz, 1/10,000) for the detection of cytoplasmic proteins and anti-acetyl-histone H3 (rabbit polyclonal, 06-599, Millipore, 1/5000) for the detection of nuclear proteins (data not shown). Finally, the two same antibodies used in the IH experiments (antibody against Extra-RAGE, mouse/rat anti-RAGE, MAB1179, R&D systems, 1/3000 and antibody against Intra-RAGE rabbit polyclonal to RAGE, ab3611, Abcam, 1/1500) were used for detection of Full-RAGE in the cytoplasmic fraction and its intracellular domain in the nuclear fraction.

**MMP9 activity**

Brain tissue was lysed using a lysis buffer (50 mM TrisHCl pH 7.5, 150 mM NaCl, 1% SDS) without any protease inhibitors or chelators in order to maintain MMP9 activity. MMP9 and MMP2 are the only gelatinases of the MMP family; therefore, their activity can be measured using fluorescently labeled gelatin. A DQ-fluorescein-conjugated gelatin kit (EnzChek® Gelatinase/Collagenase Assay Kit, Life Technology) was used following the manufacture’s protocol.

**^1^H Magnetic Resonance Spectroscopy (MRS)**

All MR measurements were carried out on a 3T MR scanner (Magnetom TimTrio, Siemens Healthcare) with a transverse electromagnetic (TEM 3000) head coil (MR Instruments, Inc). The magnetic field homogeneity was optimized by adjusting first- and second-order shims using FAST(EST)MAP^92^. In vivo 1H-MR spectra were acquired from the mPFC^36^ using a short-TE spin-echo full-intensity acquired localized single voxel spectroscopy technique (SPECIAL)^39, 40^. The following scan parameters were used: volume of interest (VOI) = 20 × 20 × 25 mm^3^, echo time/ repetition time (TE/TR) = 6/4000 ms, acquisition bandwidth = 2 kHz, number of averages = 148, and vector size = 2048. Outer volume suppression (OVS)^93^ and water suppression with variable pulse power and optimized relaxation delays (VAPOR) were applied prior to the SPECIAL localization sequence.

***Spectral quantification:*** To obtain [GABA_mPFC_] and [Glu_mPFC_], water-suppressed in vivo 1H-MR spectra were analyzed by LCModel (Stephen Provencher, Inc) as a linear combination of model spectra provided in a basis-set consisting of 20 simulated individual metabolite spectra: alanine (Ala), aspartate (Asp), phosphocreatine (PCr), creatine (Cr), γ-aminobutyric acid (GABA), glutamine (Gln), glutamate (Glu), phosphorylcholine (PCho), glycerophosphorylcholine (GPC), glutathione (GSH), glucose (Glc), lactate (Lac), glycine (Gly), myo-inositol (mIns), N-acetylaspartylglutamate (NAA), N-acetylaspartylglutamate (NAAG), ascorbate (Asc), phosphoryl-ethanolamine (PE), scylloinositol (sIns), taurine (Tau), and an experimentally measured macromolecule baseline. Unsuppressed water 1H MR spectra were used as an internal reference for the quantification of metabolite concentrations. The spectral range for analysis was set to 0.2–4.2 ppm. Tissue composition inside the MRS volume used for the water content calculation and partial volume correction was calculated based on the segmentation of magnetization-prepared rapid gradientecho (MPRAGE) images (TE/TR = 2.98/2300 ms, TI = 900 ms, flip angle = 9 degree, FOV = 240 × 256 mm^2^, matrix = 240 × 256, slice thickness = 1.2 mm) using an in-house software (more details are reported elsewhere^36^).

**Statistical analysis**

For animal experiments, data were tested to determine if they met parametric criteria, including normality of residuals and homogeneity of variances using Shapiro and Bartlett tests, respectively. As these criteria were met, parametric tests were used, such as Student’s t test, when only two groups were compared, or a two-way ANOVA, when several groups were compared with 2 different factors (e.g., genotype and treatment, or genotype and age). To further evaluate group differences, we used a Tukey post hoc test for multiple comparison tests. For human analysis, data did not pass the parametric tests (Shapiro and Bartlett tests), therefore squares of the data were used, and technical outliers were removed for the next steps, when their value were higher than values described in literature. This was the case for one control and one patient that were removed from the analysis. The influence of different factors on the sRAGE levels was tested using a linear regression model. The factors that were not significantly associated with sRAGE measurements were excluded from the analyses. Finally, a linear regression model including factors of interest was tested. The correlation between 2 different parameters was tested with a correlation test. All statistical analyses were conducted on R studio software.

***Sample size:*** Three to four randomized litters (per age and per genotype group) were used to generate the experimental WT and KO mice. When a treatment was applied (e.g SB-3CT), treated and vehicle animals were randomly taken from each litter, for internal control of the treatment. Based on previous findings, the number of animals per group and brain sections per animals were chosen to detect ~ 25% change in number of PV-IR cells and ~ 75% change in 8-oxo-dG intensity with a power of 80% at a significant α-value set to p = 0.05. To ensure the size sample used had adequate power, data was checked using the tool power details in R studio software. The threshold for conservative power was always taken above 0.7. Type 1 error was set at 0.05 (alpha) and the difference to detect between means was taken at the level of 0.7 – 0.8.

***Blinding:*** For animal data, the investigators were blind to mice genotype (WT or KO) or treatment (SB-3CT or vehicle) during material acquisition and experimental analysis, as an ID number was attributed to each mouse. A section from a control WT mouse was first used to set the image acquisition parameters of the confocal microscope. Thereafter, image acquisition for all animals was performed blindly. For Human data, the investigators were blinded to group (control or patient) allocation at the time of experimental analysis (blood collection and processing, ELISA and MRS scanning), as an ID number was attributed to all subjects.

**References**

85. Yung AR, Yuen HP, McGorry PD, Philips LJ, Kelly D, Dell’Olio M, *et al.* Mapping the onset of psychosis: the Comprehensive Assessment of At-Risk Mental States. *Aust N Z J Psychiatry.* 964-71 (2005).

86. Preisig M, Fenton BT, Matthey ML, Berney A, Ferrero F. Diagnostic interview for genetic studies (DIGS): inter-rater and test-retest reliability of the French version. *Eur Arch Psychiatry Clin Neurosci*. 249(4):174-9 (1999).

87. Kay SR, Fiszbein A, Opler LA. The positive and negative syndrome scale (PANSS) for schizophrenia. *Schizophr Bull.* 13(2):261-76 (1987).

88. Andreasen NC, Pressler M, Nopoulos P, Miller D, Ho BC. Antipsychotic dose equivalents and dose-years: a standardized method for comparing exposure to different drugs. *Biol Psychiatry*. 67(3):255-62 (2010).

89. Kulak A, Steullet P, Cabungcal JH, Werge T, Ingason A, Cuenod M, *et al*. Redox Dysregulation in the Pathophysiology of Schizophrenia and Bipolar Disorder: Insights from Animal Models. Antioxid. Redox Signal. 18, 1428–1443 (2013).

90. Dalkara, D, Byrne LC, Lee T, Hoffmann NV, Schaffer DV & Flannery JG. Enhanced gene delivery to the neonatal retina through systemic administration of tyrosine-mutated AAV9. Gene Ther 19, 176–181 (2012).

91. Gholizadeh S, Tharmalingam S, Macaldaz ME & Hampson DR. Transduction of the central nervous system after intracerebroventricular injection of adeno-associated viral vectors in neonatal and juvenile mice. Hum Gene Ther Methods 24, 205–213 (2013)

92. Gruetter, R. Automatic, localized in vivo adjustment of all first- and second-order shim coils. Magn Reson Med 29, 804–811 (1993).

93. Tkác I, Andersen P, Adriany G, Merkle H, Ugurbil K, Gruetter R. In vivo 1H NMR spectroscopy of the human brain at 7 T. Magn Reson Med 46, 451–456 (2001).
